# Colds

**Published:** 1879-04

**Authors:** P. H. Hayes


### COLDS AND THE COLD-CATCHING TENDENCY.

P. H. HAYES, M. D.

Colds are among the penalties we pay for our high civilization. Methuselah lived to the mature age of nine hundred and sixty-nine years, and it is safe to say he never had a cold in all his life. The causes of disease were in the world then even as now, but the constitutions of men were so strong to resist them, that in fact, they did not appear to be causes at all. Indeed, the race remained ignorant for ages of a thousand conditions of disease, because their constitutions were never affected by them. At the present moment, probably three-fourths of the more than thirteen hundred varieties of diseases classified by medical experts, are awakened by colds. Indeed, this seems to be just what a cold is for, to seize us at our weakest point, and to arouse in us any latent susceptibility we may have to any form of disease. Is a man of a rheumatic habit of body, then a cold will surely give him a taste of the rheumatism. Is he of a gouty constitution, then takes he a cold, presto, the gouty pains begin in his toes, and may feel their way onward and visit every joint in his body. In the same manner colds may awaken neuralgia, scrofula, and bilious disorders. But of all points of attack the most frequent and familiar are the nose, the throat and the lungs. Indeed, so much more frequently do they tell upon these organs than upon others, that all ordinary notions of colds are gotten from observing their course and symptoms in the head, throat and lungs. Is there a latent susceptibility to lung disease, asthma, bronchitis, consumption? Colds are sure to awaken it, and in time establish incurable morbid changes.

We need not stop to define a cold, as the doctors would say, pathologically, for does not every one know by experience what a cold is? The vicissitudes of climate, and the special agencies of cold and damp, are everywhere at work, and we can in general terms account for colds. But the how we got this particular cold is often a mystery. One gentleman I observed, is in the habit of taking off thick boots after the day's work is done, and putting on slippers, and thus going about his house, and even his grounds. This man has almost lost his voice from throat disease, yet his cold upon cold is to him a mystery. A lady goes out calling in the spring, or in the fall of the year; at one house she finds the parlor warm, and at the next she is kept waiting in a cold room for the mistress until she is chilled through, and she wonders the next day how she could have taken cold, as she did not take her things off once. Others breathe the warm air from a furnace, or a stove, during all the hours of sleep, and then are surprised that they cannot bear the out-door air, and that they are troubled so much of the time by hoarseness, by a stuffed up feeling in the head, and by tightness of the lungs. Those who ride much in the close atmosphere of railroad cars, and often without laying off outside wrappings, will find themselves constantly taking colds, and to them, perhaps, very mysteriously. It is not long since the journals gave us the case of a young lady, whose continued colds and neuralgia were a mystery to her physician, until he made the discovery that she was in the habit of reading and writing with her arms resting on a marble-top table. A lady follows her callers to the door, and there, without any protection for herself, waits to say and to hear so many last things, that she finally retreats with a shiver, but when her cold sets in, she cannot imagine how she has taken it.

The cold-catching tendency is created by all such habits as weaken the power of the body to create its own vital warmth. All persons of feeble, heat-producing power, take cold easily. In this respect we are creatures of habit. By so much as we get in the habit of supplying artificial heat to our bodies, by so much are we the more liable to take cold. Ladies who sit with feet on stove or register, and take hot flat-irons or soapstone to bed with them, will soon find they can hardly go from room to room in the house without catching cold, and as to going out of doors, that is hardly to be thought of. Business men often sit too near stoves in their offices, and the warmth relaxes the skin, opens the pores, and on going into the air they are immediately chilly. It is a very common habit to sit in church or at a concert, and worst of all in railway coaches, with all the upper garments on, and this habit so weakens the skin as to establish the cold-catching tendency. The free use of hot tea and coffee, or other hot drinks, does unquestionably predispose to cold-catching. The frequent use of the warm bath must also be classed in the same category. Bundling the neck and face is a sure way of predisposing to colds in the head and throat.

The true philosophy of curing the cold-catching habit is to educate the body to bear all ordinary and inevitable exposures without harm. Begin by walking the body warm twice a day in the open air. This improves appetite and digestion, promotes an increase of blood, warmth and electricity, and by so much a powerful resistance to cold and damp and all the ordinary conditions of cold-taking. In all those countries where the people live much in the open air, in the South of Europe, where windows are almost unknown in their houses, colds, and the diseases caused by colds, are also almost unknown. The salt towel bath taken on retiring or on rising is one of the surest ways of defending the system against colds. It should always be taken in a comfortably warmed room, and at about eighty degrees.

As to the modes of curing a fresh cold, aconite in drop doses is the best of all in the feverish stage. Inhalations of ethereal tincture of camphor are also extremely beneficial. The emulsion of carbonate of ammonia is very highly recommended in the very first stages by Dr. Dobell, of London. Almost any of the essential oils, taken in drop doses in dilute, solution or emulsion, at intervals of one to two hours, will aid greatly in breaking up a cold at any stage. But these suggestions are intended for the physician, and not for the patient to follow without consulting his medical adviser.

